# Genetic analysis redraws the management boundaries for the European sprat

**DOI:** 10.1111/eva.12942

**Published:** 2020-03-17

**Authors:** María Quintela, Cecilie Kvamme, Dorte Bekkevold, Richard D. M. Nash, Eeva Jansson, Anne Grete Sørvik, John B. Taggart, Øystein Skaala, Geir Dahle, Kevin A. Glover

**Affiliations:** ^1^ Institute of Marine Research Bergen Norway; ^2^ DTU‐Aqua National Institute of Aquatic Resources Technical University of Denmark Silkeborg Denmark; ^3^ Institute of Aquaculture School of Natural Sciences University of Stirling Stirling UK; ^4^ Institute of Biology University of Bergen Bergen Norway

**Keywords:** ddRADseq, fisheries, management, population structure, SNPs, *Sprattus sprattus*

## Abstract

Sustainable fisheries management requires detailed knowledge of population genetic structure. The European sprat is an important commercial fish distributed from Morocco to the Arctic circle, Baltic, Mediterranean, and Black seas. Prior to 2018, annual catch advice on sprat from the International Council for the Exploration of the Sea (ICES) was based on five putative stocks: (a) North Sea, (b) Kattegat–Skagerrak and Norwegian fjords, (c) Baltic Sea, (d) West of Scotland—southern Celtic Seas, and (e) English Channel. However, there were concerns that the sprat advice on stock size estimates management plan inadequately reflected the underlying biological units. Here, we used ddRAD sequencing to develop 91 SNPs that were thereafter used to genotype approximately 2,500 fish from 40 locations. Three highly distinct and relatively homogenous genetic groups were identified: (a) Norwegian fjords; (b) Northeast Atlantic including the North Sea, Kattegat–Skagerrak, Celtic Sea, and Bay of Biscay; and (c) Baltic Sea. Evidence of genetic admixture and possibly physical mixing was detected in samples collected from the transition zone between the North and Baltic seas, but not between any of the other groups. These results have already been implemented by ICES with the decision to merge the North Sea and the Kattegat–Skagerrak sprat to be assessed as a single unit, thus demonstrating that genetic data can be rapidly absorbed to align harvest regimes and biological units.

## 1 INTRODUCTION

Increasing global attention is being given to sustainable production and harvest of human food from the marine environment. This is occurring at a time when many of the world's fisheries are either overexploited, depleted, or recovering from earlier depletion (FAO, 2016), challenges with illegal, unreported, and unregulated fishing (IUU) are extensive (Agnew et al., 2009), and the degree of climate‐driven changes in many of the world's marine ecosystems is unparalleled (Frainer et al., 2017; Stige & Kvile, 2017). Consequently, there is a growing need to develop tools that ensure the sustainable management of the living marine resources.

Failing to take the underlying components of fisheries into consideration, such as the spatiotemporal mixing of populations, can lead to differential exploitation and potential overexploitation of resources (Allendorf, England, Luikart, Ritchie, & Ryman, 2008; Kerr et al., 2017). Although genetic data for some marine fish species have existed for decades (Hauser & Carvalho, 2008), their application in fisheries management was initially slow (Reiss, Hoarau, Dickey‐Collas, & Wolff, 2009; Waples, Punt, & Cope, 2008). However, genetic and genomic methods are now providing unprecedented levels of precision in understanding connectivity among marine populations (Besnier et al., 2014; Dahle, Quintela, et al., 2018; Hemmer‐Hansen et al., 2019), and in many cases have led to increased understanding of potential mechanisms underlying local adaptation (Ayllón et al., 2015; Kirubakaran et al., 2016; Martínez Barrio et al., 2016). The ICES Stock Identification Methods Working Group (SIMWG) reviews new approaches for stock identification with genetic techniques as one of its core methodologies. Recommendations on the validity and use of results from the various stock identification techniques are given to the relevant working groups for use in their stock assessments. Genetic and genomic tools have been applied directly to management issues, including “real‐time” regulation of harvest (Dahle, Johansen, Westgaard, Aglen, & Glover, 2018; Johansen et al., 2018), cost‐effective fisheries enforcement (Glover, 2010; Martinsohn et al., 2019), and updated management plans (Mullins, McKeown, Sauer, & Shaw, 2018; Saha et al., 2017; Westgaard et al., 2017). The definition of stock units in fisheries management needs to consider the spatial structure of biological populations to prevent overexploitation of unique spawning components. There is the general recognition, at least within the Northeast Atlantic, that this is one the main threats to sustainable fisheries, with recent studies also highlighting other problems and suggesting ways to act accordingly (see Kerr et al., 2017 for revision).

The European sprat, *Sprattus sprattus* (L.), hereafter referred to as sprat, is a fast‐growing, small, short‐lived pelagic shoaling fish (Moore et al., 2019; Peck et al., 2012) inhabiting the Northeast Atlantic from northern Norway to Morocco and into the Baltic Sea, the northern Mediterranean basins, and the Black Sea (Debes, Zachos, & Hanel, 2008). Sprat has formed the basis for a fishery throughout most of its natural distribution, and it is also an important prey for different piscivorous fishes, marine mammals, and seabirds (ICES, 2013, 2018d). The International Council for the Exploration of the Sea (ICES, www.ices.dk) provides annual catch advice for this species. The management of exploitation, specifically within the majority of the ICES Greater North Sea Ecoregion (ICES, 2018a, 2018b), consists of an “escapement strategy” whereby the aim is to maintain the stock above a certain critical level by using an upper limit (cap) on fishing mortality (*F*_cap,_ currently set at 0.7). The sprat abundance assessment uses a natural mortality estimate derived from a multispecies model including many of its predators, thus partly ensuring a exploitation level, which will not negatively impact populations reliant on sprat as a prey source (ICES, 2013, 2018c). The total catch (commercial harvest) can therefore vary quite considerably interannually depending on the strength of an incoming year class (see ICES, 2018e). In the Norwegian fjords, sprat catches have declined from ~18,000 tonnes in 1973 to ~1,315 tonnes in 2018 (source: Directorate of Fisheries, Norway). Although the causative reasons for the declining catches are not fully known, they partly reflect a reduction in abundance as well as vessels participating in this fishery.

Sprat displays population genetic structure throughout its distribution (Debes et al., 2008; Glover, Skaala, Limborg, Kvamme, & Torstensen, 2011; Limborg, Hanel, et al., 2012; Limborg, Pedersen, Hemmer‐Hansen, Tomkiewicz, & Bekkevold, 2009). For example, genetic differences have been observed among sprat sampled in the Norwegian fjords, the North and the Baltic Seas (Glover et al., 2011), and between samples from the Baltic Sea and the Kattegat–Skagerrak area (Limborg et al., 2009). No clear differentiation has been identified between populations spawning east and west of the British Isles (Limborg et al., 2009). However, these previous studies were based on mtDNA or fewer than ten microsatellite DNA markers, and although they have provided some knowledge of genetic structure especially in the Northeast Atlantic, more rigorous tools, such as those incorporating more loci and/or full‐genome coverage, are often needed to obtain enough resolution for determining local‐scale processes in marine fish populations (e.g., Bekkevold et al., 2015b; Carreras et al., 2017; Figueras et al., 2016; Tine et al., 2014).

Until recently, ICES provided advice on maximum total catch on five separate sprat stocks in the Northeast Atlantic: (a) North Sea, (b) Kattegat–Skagerrak and Norwegian fjords, (c) Baltic Sea, (d) West of Scotland—southern Celtic Sea, and € English Channel (ICES, 2013). However, this stock delineation was considered as unlikely to adequately reflect the true underlying biological units, that is, populations. Consequently, there was a stated need to improve the knowledge about population genetic structure to describe the biological units in order to inform more sustainable exploitation (ICES, 2018c, 2018f, 2019). In the present study, we addressed this issue by performing a genetic analysis of an extensive set of sprat sampled in the North Sea, Kattegat–Skagerrak, and Baltic Sea areas, with the aim to strengthen input to harvest advice and management. We also analyzed samples from a substantial number of Norwegian fjord systems spanning around 1,560 km, to infer demographics of these units. In order to achieve this, we first identified single nucleotide polymorphism markers (SNPs) throughout the genome by using ddRAD sequencing, and thereafter genotyped and analyzed approximately 2,500 sprat from the geographical areas described.

## 2 MATERIALS AND METHODS

### 2.1 Sampling

**Figure 1:**
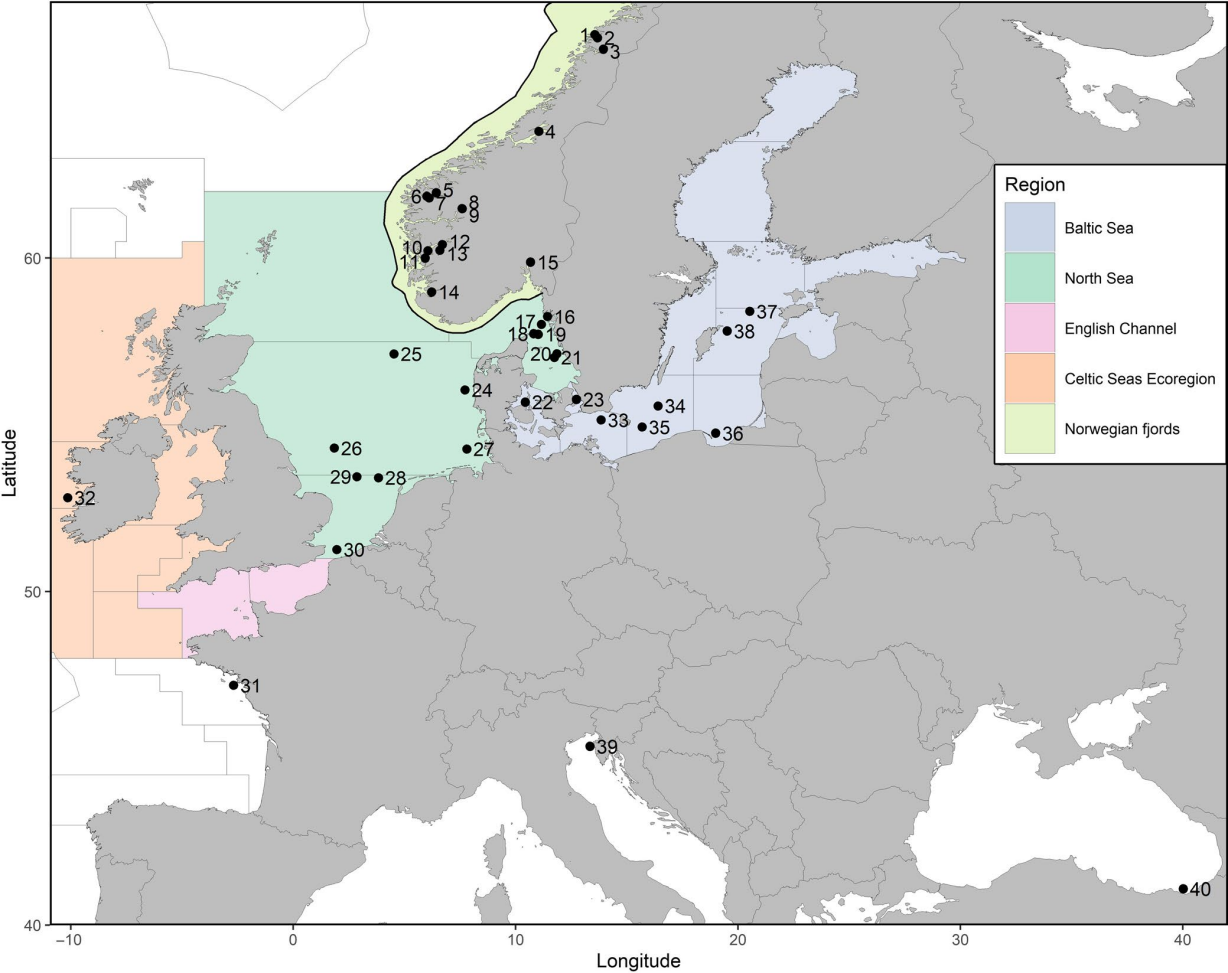
Map of the sampling locations. The colored areas show the different management areas used by ICES for giving advice in 2019. Codes and associated full names for the sampling sites can be found in Table 1

Approximately 2,500 sprat were sampled by commercial fishermen and scientific cruises from forty locations in the NE Atlantic (Figure 1). Part of these samples had been formerly analyzed using microsatellite markers in previous studies (see Table 1). As there is a strong management interest in defining stock affiliation of sprat fished in the Kattegat–Skagerrak areas, sprat were sampled in these areas both during the spring spawning season and outside the spawning season by the pelagic fishery. Norwegian fjord samples spanning most of the sprat's Norwegian distribution range were also sampled. To compare with geographically more distant populations, samples were included from the Bay of Biscay, the Celtic Sea, and two out‐groups representing the southernmost distribution of the species: the Adriatic Sea and the Black Sea. Sample size per location ranged from 21 to 116 individuals. Sprat are indeterminate batch spawners (i.e., individual fish may spawn over protracted periods) and locally the spawning season may stretch over the majority of the year (e.g., Ojaveer & Kalejs, 2010). Sampling spawning individuals represents the most robust approach to delineating population genetic structure and sampling was directed toward ripe individuals, where possible. However, in some areas (Table 1), samples were mainly taken outside the main spawning season and may thus represent both local and migratory individuals of mixed origin.

**Table 1:** Sample summary statistics: Sampling site within geographic regions (code of the site and number in the map), time of collection (year/month), coordinates, number of individuals (No ind); observed heterozygosity, Ho (mean ± *SE*); unbiased expected heterozygosity, uHe (mean ± *SE*); inbreeding coefficient, *F*_IS_ (mean ± *SE*); number of deviations from Hardy–Weinberg equilibrium (HWE) at *α* = .05; and number of deviations from linkage disequilibrium (LD) at *α* = .05 both before and after (B) Bonferroni correction. Samples from ripe individuals are depicted in boldface type in the year/month columns. Samples collected and analyzed in connection with previous microsatellite marker studies (1. Limborg et al., 2009, 2. Glover et al., 2011, 3. Limborg, Hanel, et al., 2012) are indicated

Region	Code	No	Year	Month	Site	Latitude	Longitude	No ind	Ho	uHe	F_IS_	No dev HWE (B)	No dev LD (B)	Study
Norwegian fjords	HOL	1	2008	12	Holandsfjord	66.71	13.63	31	0.278 ± 0.018	0.282 ± 0.016	0.003 ± 0.021	7 (0)	126 (1)	2
	MEL	2	2008	12	Melfjord	66.61	13.58	79	0.286 ± 0.016	0.281 ± 0.015	−0.021 ± 0.013	7 (0)	193 (2)	2
	FIN	3	2008	12	Finneidfjord	66.21	13.81	75	0.276 ± 0.015	0.286 ± 0.015	0.016 ± 0.015	8 (0)	178 (2)	2
	TRH	4	2008	12	Stjørdalsfjord	63.47	10.86	80	0.281 ± 0.015	0.286 ± 0.015	0.005 ± 0.013	5 (1)	193 (4)	2
	NOR1	5	2015	12	Nordfjord	61.96	06.43	39	0.275 ± 0.017	0.286 ± 0.016	0.024 ± 0.020	7 (2)	141 (2)	
	NOR2	6	**2001**	**5**	Nordfjord	61.85	05.85	74	0.284 ± 0.017	0.286 ± 0.016	0.008 ± 0.014	7 (0)	196 (5)	2
	NOR3	7	2015	12	Nordfjord	61.81	06.11	49	0.264 ± 0.016	0.283 ± 0.016	0.053 ± 0.019	7 (3)	153 (2)	
	SOG1	8	2008	11	Sognefjorden	61.49	07.59	47	0.264 ± 0.016	0.278 ± 0.016	0.024 ± 0.018	10 (1)	156 (2)	2
	SOG2	9	2015	12	Sognefjorden	61.48	07.59	116	0.266 ± 0.014	0.283 ± 0.015	0.047 ± 0.014	16 (2)	202 (4)	
	HAR1	10	2015	12	Hardangerfjorden	60.22	06.05	100	0.269 ± 0.014	0.283 ± 0.015	0.032 ± 0.010	7 (0)	203 (4)	
	HAR2	11	2008	11	Hardangerfjorden	59.74	05.56	77	0.269 ± 0.015	0.284 ± 0.016	0.035 ± 0.016	12 (2)	173 (3)	2
	HAR3	12	2008	11	Hardangerfjorden	60.41	06.67	46	0.286 ± 0.016	0.284 ± 0.015	−0.018 ± 0.017	5 (0)	160 (2)	2
	HAR4	13	2008	11	Hardangerfjorden	60.14	06.56	99	0.278 ± 0.015	0.282 ± 0.015	0.004 ± 0.013	9 (3)	188 (3)	2
	LYS	14	2008	11	Lysefjorden	58.92	06.09	100	0.273 ± 0.015	0.286 ± 0.016	0.032 ± 0.013	7 (1)	176 (4)	2
	OSL	15	2007	9	Oslofjorden	59.89	10.59	89	0.269 ± 0.016	0.281 ± 0.016	0.034 ± 0.013	7 (1)	209 (2)	2
Kattegat–Skagerrak	UV	16	**2008**	**5**	Uddevalla fjord	58.12	11.52	59	0.232 ± 0.017	0.244 ± 0.018	0.023 ± 0.016	6 (1)	134 (2)	3
	SK1	17	2018	6	Kattegat	58.01	11.15	58	0.231 ± 0.019	0.239 ± 0.019	0.017 ± 0.016	6 (1)	114 (1)	
	SK2	18	**2006**	**3**	Kattegat	57.42	10.48	38	0.227 ± 0.020	0.227 ± 0.019	−0.004 ± 0.019	5 (1)	82 (1)	3
	SK3	19	2018	9	Kattegat	57.71	11.01	38	0.211 ± 0.018	0.239 ± 0.019	0.107 ± 0.026	16 (3)	139 (1)	
	SK4	20	2018	7	Kattegat	57.13	11.85	41	0.218 ± 0.018	0.230 ± 0.019	0.021 ± 0.018	3 (1)	102 (1)	
	SK5	21	2018	7	Kattegat	57.02	11.74	73	0.228 ± 0.019	0.241 ± 0.019	0.044 ± 0.018	9 (2)	126 (2)	
	GB	22	**2006**	**3**	Great Belt	55.42	10.25	47	0.251 ± 0.018	0.254 ± 0.017	0.005 ± 0.016	4 (0)	135 (1)	1, 3
	ØS	23	**2006**	**3**	Øresund	55.76	12.73	46	0.263 ± 0.019	0.257 ± 0.018	−0.025 ± 0.017	5 (0)	129 (1)	3
Atlantic/North Sea	NS1	24	2018	7	North Sea	56.04	07.72	57	0.214 ± 0.018	0.231 ± 0.018	0.082 ± 0.024	11 (5)	158 (6)	
	NS2	25	**2015**	**5**	North Sea	57.13	04.52	77	0.231 ± 0.018	0.238 ± 0.018	0.008 ± 0.013	3 (1)	135 (1)	
	NS3	26	2008	1	North Sea	54.307	01.84	93	0.233 ± 0.018	0.246 ± 0.018	0.029 ± 0.014	9 (2)	137 (1)	2
	NS4	27	**2005**	**5**	North Sea	55.40	06.46	59	0.224 ± 0.019	0.235 ± 0.019	0.042 ± 0.018	9 (2)	127 (1)	3
	NS5	28	2016	8	North Sea	53.41	03.83	40	0.229 ± 0.019	0.234 ± 0.019	0.027 ± 0.020	7 (2)	125 (1)	
	NS6	29	2016	8	North Sea	53.44	02.85	38	0.231 ± 0.018	0.241 ± 0.019	0.014 ± 0.017	4 (1)	98 (1)	
	EC	30	**2009**	**6**	English Channel	51.14	01.57	50	0.218 ± 0.018	0.228 ± 0.019	0.019 ± 0.016	7 (1)	108 (2)	3
	BoB	31	2008	8	Bay of Biscay	47.40	−02.38	57	0.214 ± 0.018	0.234 ± 0.019	0.085 ± 0.023	12 (4)	110 (2)	3
	CEL	32	2009	10	Celtic Sea	52.80	−10.08	79	0.242 ± 0.018	0.245 ± 0.019	−0.007 ± 0.013	5 (1)	123 (1)	2
Baltic Sea	AB	33	**2006**	**5**	Arkona Basin	55.08	13.50	59	0.232 ± 0.018	0.237 ± 0.019	0.006 ± 0.015	5 (1)	125 (1)	1, 3
	BBN	34	**2006**	**3**	Bornholm Basin *N*	55.34	16.25	39	0.224 ± 0.020	0.226 ± 0.019	−0.016 ± 0.016	4 (0)	103 (1)	1, 3
	BBS	35	**2006**	**3**	Bornholm Basin S	55.13	16.14	43	0.238 ± 0.020	0.238 ± 0.019	−0.014 ± 0.017	6 (0)	90 (1)	1, 3
	GD	36	**2006**	**3**	Gdańsk Deep	54.43	18.60	56	0.227 ± 0.019	0.238 ± 0.019	0.036 ± 0.019	6 (2)	119 (1)	1, 3
	GOTB	37	**2006**	**5**	Gotland Basin	58.24	20.31	55	0.225 ± 0.019	0.232 ± 0.019	0.007 ± 0.015	5 (0)	130 (1)	2
	GOT	38	**2006**	**3**	Gotland	58.24	20.31	56	0.240 ± 0.022	0.223 ± 0.019	−0.053 ± 0.017	8 (0)	110 (1)	1, 3
Adriatic Sea	ASA	39	2005	12	Adriatic Sea	45.36	13.34	45	0.179 ± 0.018	0.195 ± 0.020	0.050 ± 0.021	8 (3)	75 (0)	1, 3
Black Sea	BS	40	2008	12	Black Sea	41.05	40.00	21	0.193 ± 0.020	0.208 ± 0.020	0.036 ± 0.027	9 (0)	47 (0)	3

### 2.2 SNP isolation and genotyping

DNA was extracted from fin clips stored in ethanol using the Qiagen DNeasy 96 Blood & Tissue Kit in 96‐well plates, each of which contained two or more negative controls.

A double‐digest RAD library was constructed from eight sprat genomic DNA samples from Hardangerfjorden, comprising a 400–700 base pair region of *Sbf*I‐ and *Sph*I‐restricted DNA and involving individual‐specific inline barcode adapters. The methodology has been previously described in detail by Manousaki et al. (2015). The library was thereafter sequenced on the Illumina MiSeq platform (part of a shared flow cell run, V2 chemistry, 300 cycle kit, 160 base paired‐end reads). Stacks software v1.47 (Catchen, Hohenlohe, Bassham, Amores, & Cresko, 2013) was used to demultiplex sequence reads and identify and score SNPs (de novo assembly; key Stacks parameters m (minimum depth of coverage) = 4, *M* (maximum distance allowed between stacks) = 2, *n* (number of mismatches allowed between loci among individuals) = 1). Data were then exported to Microsoft Excel for filtering to identify potential SNPs suitable for Sequenom‐based multiplex SNP assay. This involved selecting RAD loci (trimmed length 135 bases) that contained a single diallelic SNP with at least two occurrences of the minor allele among the eight samples and that the SNP was positioned between base 41 and base 95, to allow for enough flanking sequence for PCR primer design. For the final filtered set, SNP locus primer design, amplification, and genotype calling were performed using the Sequenom MassARRAY iPLEX Platform, as described by Gabriel, Ziaugra, and Tabbaa (2009).

### 2.3 Statistical analysis

Observed heterozygosity (*H*_o_) and unbiased expected heterozygosity (*uH*_e_), as well as the inbreeding coefficient (*F*_IS_), were computed for each sample with GenAlEx (Peakall & Smouse, 2006). The genotype frequencies of each locus and its direction (heterozygote deficit or excess) were compared with Hardy–Weinberg expectations (HWEs) using the program GENEPOP 7 (Rousset, 2008) as was linkage disequilibrium (LD) between pairs of loci. HWE and LD were examined using the following Markov chain parameters using 10,000 steps of dememorization, 1,000 batches, and 10,000 iterations per batch, and signification was assessed after the *post hoc* sequential Bonferroni correction (Holm, 1979).

Many marine fish species display a weak genetic population structure because populations are large and gene flow is high (Ward, Woodwark, & Skibinski, 1994). As a consequence, the majority of genetic markers may be uninformative about demographic processes, which has fueled the search for loci carrying signatures of locally divergent selection that might serve as powerful markers to assess spatially explicit genetic structure as well as to outline stocks for fisheries management (Russello, Kirk, Frazer, & Askey, 2012). Here, loci deviating from neutrality were statistically identified using two complementary outlier approaches: the hierarchical Bayesian method described in Beaumont and Balding (2004) and implemented in BayeScan software (Foll & Gaggiotti, 2008), and the Fdist approach of Beaumont and Nichols (1996) implemented in LOSITAN (Antao, Lopes, Lopes, Beja‐Pereira, & Luikart, 2008). To minimize the risk of detecting false positives, only the putative outliers flagged by both procedures were retained. BayeScan was run by setting sample size to 10,000 and the thinning interval to 50 as suggested by Foll and Gaggiotti (2008). The loci with a posterior probability above 0.99 were retained as outliers, corresponding to a Bayes factor >2, that is, “decisive selection” (Foll & Gaggiotti, 2006). In LOSITAN, a neutral distribution of *F*_ST_ with 100,000 iterations was simulated, with a forced mean *F*_ST_ at a significance level of 0.05 under an infinite allele model. Under both approaches, the outlier tests were conducted in two different ways: (a) including all the locations in the same analysis, both excluding and including the southern out‐group samples (i.e., 38 and 40 samples, respectively), and (b) in a pairwise manner between regions. In the pairwise analysis, all the fish sampled within a region (e.g., Norwegian fjords and Baltic Sea; see Table 1) were pooled together into a single “sample” from which a random subset of individuals was extracted. The number of individuals per sample in the pairwise design was kept identical to avoid bias due to uneven sample size.

Population genetic structure was examined by estimating *F*_ST_ (Weir & Cockerham, 1984) between sample pairs using ARLEQUIN v.3.5.1.2 (Excoffier, Laval, & Schneider, 2005). Statistical significance was calculated after 10,000 permutations followed by sequential Bonferroni correction. The Bayesian clustering approach implemented in STRUCTURE v. 2.3.4 (Pritchard, Stephens, & Donnelly, 2000) was used to identify genetic groups under a model assuming admixture and correlated allele frequencies and no population prior. The analysis was conducted using the program ParallelStructure (Besnier & Glover, 2013) which distributes STRUCTURE runs among parallel processors to speed up the computational time. Ten runs with a burn‐in period consisting of 100,000 replications and a run length of 1,000,000 MCMC iterations were performed for *K* = 1 to *K* = 10 clusters. To determine the number of genetic groups in the data, STRUCTURE output was analyzed using two approaches. Firstly, the ad hoc summary statistic ΔK of Evanno, Regnaut, & Goudet (2005) was calculated. Secondly, StructureSelector (Li & Liu, 2018) was used to estimate four alternative statistics (MedMed, MedMean, MaxMed, and MaxMean), which have been described as more accurate than the previously used methods to determine the best fit number of clusters, for both even and uneven sampling data. Finally, the ten runs for the selected Ks were averaged with CLUMPP v.1.1.1 (Jakobsson & Rosenberg, 2007) using the FullSearch algorithm and the G’ pairwise matrix similarity statistic, and graphically displayed using barplots. Genetic clustering was also examined and visualized using discriminant analysis of principal components (Jombart, Devillard, & Balloux, 2010) in *adegenet* (Jombart, 2008).

Kattegat‐Skagerrak is known to be a hybrid zone for a number of marine taxa (e.g., Luttikhuizen, Drent, Peijnenburg, Veer, & Johannesson, 2012; Nielsen, Hansen, Ruzzante, Meldrup, & Grønkjær, 2003; Väinölä & Hvilsom, 2008). To elucidate the potential mixing and interaction between North Sea and Baltic Sea sprat in the Kattegat–Skagerrak contact zone, given the strong interest in defining stock affiliation and allocation of individuals back to their respective stock of sprat fished in this area, a set of 150 in silico simulated individuals was created by HYBRIDLAB (Nielsen, Bach, & Kotlicki, 2006). Parental stocks were defined by randomly selecting 150 individuals from the North Sea sites and 150 from the Baltic Sea, respectively. The set of F1 hybrids together with the parental stocks were analyzed via STRUCTURE as described above. In addition, the individual assignment option in ONCOR (Kalinowski, 2007) was used to estimate the probability of assignment of the individuals from the contact zone to the each of the three main geographic areas: Norwegian fjords, Baltic Sea, and North Sea.

To examine demographic relationships between geographically explicit samples, the genetic distance, measured as *F*_ST_/(1−*F*_ST_), between the northernmost sample (HOL, Holandsfjord) and each of the remaining ones excluding the southern European outliers, was plotted against the corresponding shortest water distance, which was calculated using the path function in GoogleEarth. The southern out‐groups were excluded for clarity as well as to limit this analysis to the samples for which management advice was intended.

## 3 RESULTS

The MiSeq run generated over 6 million paired‐end (pe) reads (1.14–2.14 pe reads per individual) identifying 5,648 rad loci. Of these, 121 putative SNPs were selected for a Sequenom‐based high‐throughput genotyping assay for screening all samples, and 99 were distributed into four multiplex reactions. After purging those SNPs for which allele discrimination was not reliable (i.e., poor clustering) as well as the ones that produced no genotypes or amplified in a very limited number of individuals (i.e., deficient amplification), 91 loci were retained for analyses. Additionally, individuals with >30% missing genotypes were purged from the dataset. The SNP loci and their corresponding flanking regions, together with the raw data, are available in Supplementary Information_Raw data, Tables S1 and S2, respectively.

The final screened data set consisted of 2,425 individuals genotyped for 91 SNPs using the Sequenom MassARRAY iPLEX Platform, 98.4% of which showed <10% of missing data. Deviations from HWE were found in 293 of the 3,640 loci by population tests (8.05%), which dropped to 51 tests (1.4%) after Bonferroni sequential correction (Table 1). The 24 loci involved were scattered across 28 out of the 40 samples and thus did not reflect any specific locus or population‐relevant signal. Out of the 163,800 performed tests for LD, 5,528 (3.4%) showed significant LD, which dropped to 74 (0.04%) after Bonferroni correction. Therefore, no loci were removed from the 91 SNP dataset.

LOSITAN analysis excluding the southern out‐groups reported thirteen candidate loci for divergent selection (14%), whereas BayeScan flagged ten (9%), all of which overlapped with those from LOSITAN (Ssp210, Ssp222, Ssp225, Ssp226, Ssp248, Ssp263, Ssp264, Ssp290, Ssp305, and Ssp319). However, when the Black and Adriatic Seas were included, LOSITAN revealed fifteen candidates of directional selection (16%) conversely to the 12 (13%) found by BayeScan. In this case, the consensus between LOSITAN and BayeScan was met for ten loci (Ssp210, Ssp222, Ssp225, Ssp226, Ssp243, Ssp248, Ssp263, Ssp264, Ssp275, and Ssp305), eight of which overlapped with the ones formerly found without the out‐groups. LOSITAN‐pairwise analyses conducted between regions after excluding the southern out‐groups revealed 1–9 loci putatively under directional selection per comparison (21 unique loci; none of them shared in all six pairwise tests). However, the consensus set incorporating BayeScan results reduced the number of outliers to three (Ssp210, Ssp263, and Ssp248). In the pairwise analyses including the southern out‐groups, no locus flagged as an outlier candidate by LOSITAN was confirmed by BayeScan.

**Figure 2:**
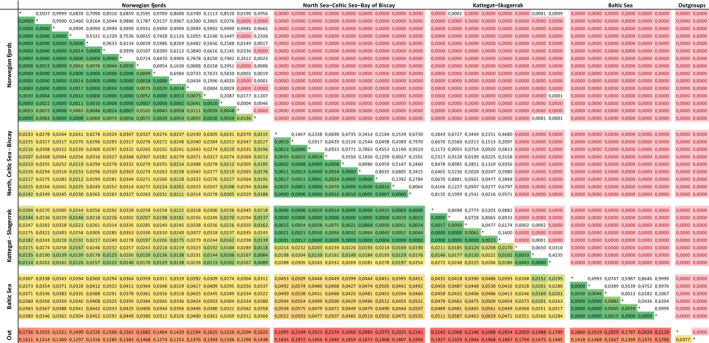
Heat map of pairwise *F*_ST_ values in the bottom diagonal and the corresponding *p*‐values after 10,000 permutations in the top diagonal. Cells highlighted in pink depict values significantly different from zero after sequential Bonferroni correction. Greener colors indicate low differentiation (*F*_ST_ closer to zero), increasing toward red to indicate large differentiation. This matrix has also been included in the Supplementary Information to ease reading

**Figure 3:**
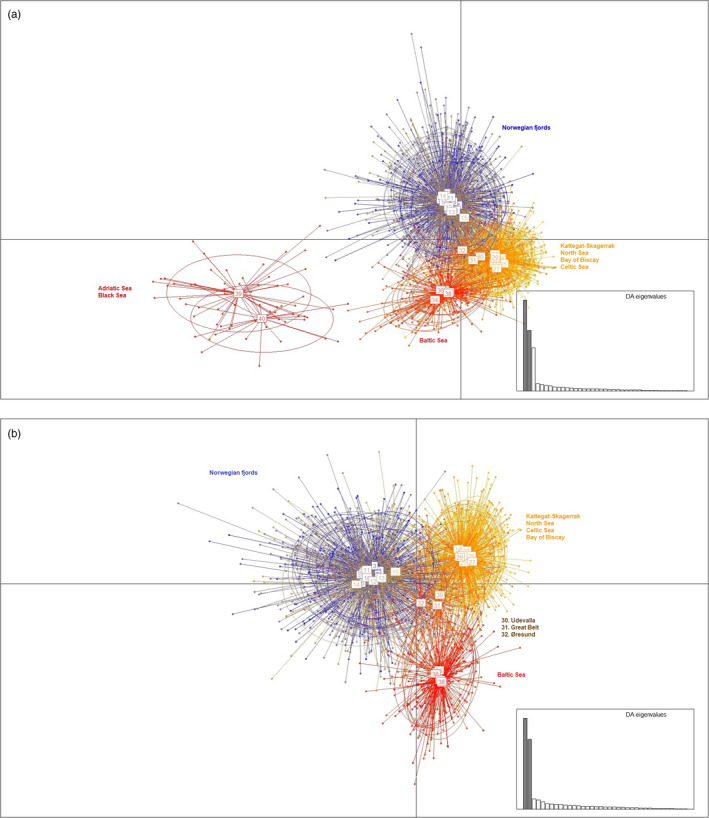
Discriminant analysis of principal components (DAPCs) for sprat samples including (a) and excluding (b) the out‐groups (i.e., Adriatic Sea and Black Sea)

Pairwise *F*_ST_ estimates ranged between 0 and 0.217, with the largest estimates found between either of the southern out‐group samples and any northern collection (ranging 0.125–0.217). The lowest estimates were found among samples within each of the geographical areas: Norwegian fjords, Baltic Sea, and North Sea–Kattegat–Skagerrak, although 9–12 pairwise comparisons within these areas still came out as statistically significant (Figure 2, Table S3). A distinct clustering of samples by geographical region was also evident in the DAPC analysis, where the first principal component (PC1), explaining 33.7% of the variation, revealed a major differentiation between the southern and northern samples (Figure 3a). PC2, accounting for 22.5% of the variation, separated samples into three main clusters: (a) Norwegian fjords, (b) Kattegat–Skagerrak–North Sea, and (c) Baltic Sea and out‐groups. Samples from the North Sea–Baltic Sea transition area Uddevalla, Great Belt, and Øresund, occupied an intermediate position without fully integrating with any of the three clusters. Samples from the Kattegat–Skagerrak area all grouped with the North Sea, irrespective of the time they were collected (during or out the spawning season). PC3 incorporated a relatively small proportion of the variation (2.8%) and mostly separated the Adriatic Sea from the other samples (Figure S1 in Supplementary Information). Lower level PCs 4–80 only explained minor degrees of variation and were not examined further. When removing the two southern out‐groups (Figure 3b), almost all variation was retained by PC1 and 2, explaining 35 and 27%, respectively. Again, the three main aforementioned regional clusters were clearly identified, and the three samples from the transition area formed an intermediate cluster between the North Sea and Baltic Sea samples. PC1 was driven mainly by ten loci, of which a single (non‐outlier) locus Ssp275 contributed twice as much as the second ranked locus, whereas PCs 2–3 were driven by several loci (Figure S2a‐c in Supplementary Information).

**Figure 4:**
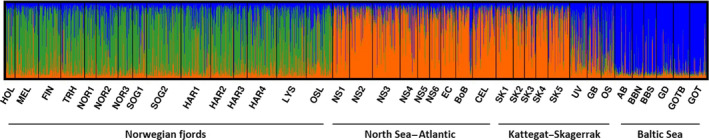
Barplot representing the proportion of individuals’ ancestry to cluster at *K* = 3 as inferred from Bayesian clustering in STRUCTURE using the total set of 91 SNP loci

**Figure 5:**
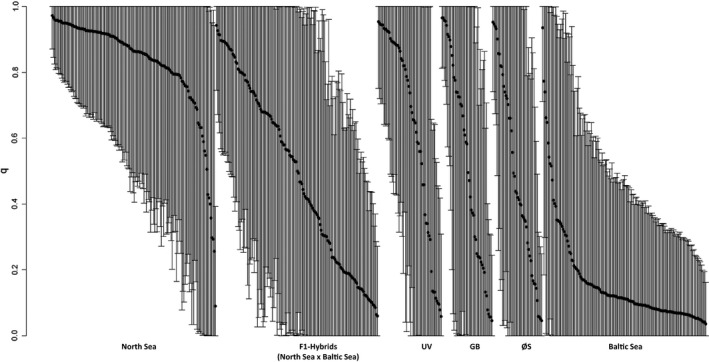
Distribution of *q* (admixture proportion) values and 90% posterior probability intervals among individuals. Samples correspond to a random suite of 150 individuals from the North Sea locations, 150 random individuals from the Baltic Sea sites, and the F1 hybrids in silico‐obtained from these two groups, and all the individuals sampled in Uddevalla (UV), Great Belt (GB), and Øresund (ØS)

In the STRUCTURE analysis, the Evanno test conducted a posteriori reported *K* = 2 as the most likely number of clusters (Δ*K* = 180.1) revealing strongest genetic divergence between Norwegian fjord sprat and all other locations. In contrast, three of the four estimators of StructureSelector reported *K* = 3 as the most likely number of genetic clusters (Figure 4), grouping samples into the same three groups as identified with DAPC. At *K* = 3, samples from Uddevalla (UV), Great Belt (GB), and Øresund (ØS) showed admixed North Sea–Baltic Sea genetic profiles with slightly higher admixture with the Baltic Sea cluster than with the North Sea cluster. An analogous pattern was also observed in the North Sea x Baltic Sea in silico‐generated hybrids (Figure 5). Thus, the plot of individual proportion of admixture (q) revealed that 80% of the in silico‐created hybrids showed overlapping confidence intervals, close to the 73% that was recorded for the true individuals in UV, GB, and ØS. In addition, the individuals showing non‐admixed profiles (either natural genotypes or created in silico) grouped with the North Sea and the Baltic Sea cluster in relatively even proportions. The exception to this was UV, in which 78% of the individuals clustered with the geographically closer North Sea group. ONCOR showed that the probabilities obtained for assignment of the individuals sampled in UV, GB, and ØS to the three main genetic clusters reflected, to a large extent, the inferred ancestry of the individuals in STRUCTURE (see Figure S3). Finally, even when the ten outlier loci were excluded from the STRUCTURE and DAPC analyses, the overall pattern revealing three distinct genetic clusters was retained (Figure S4).

**Figure 6:**
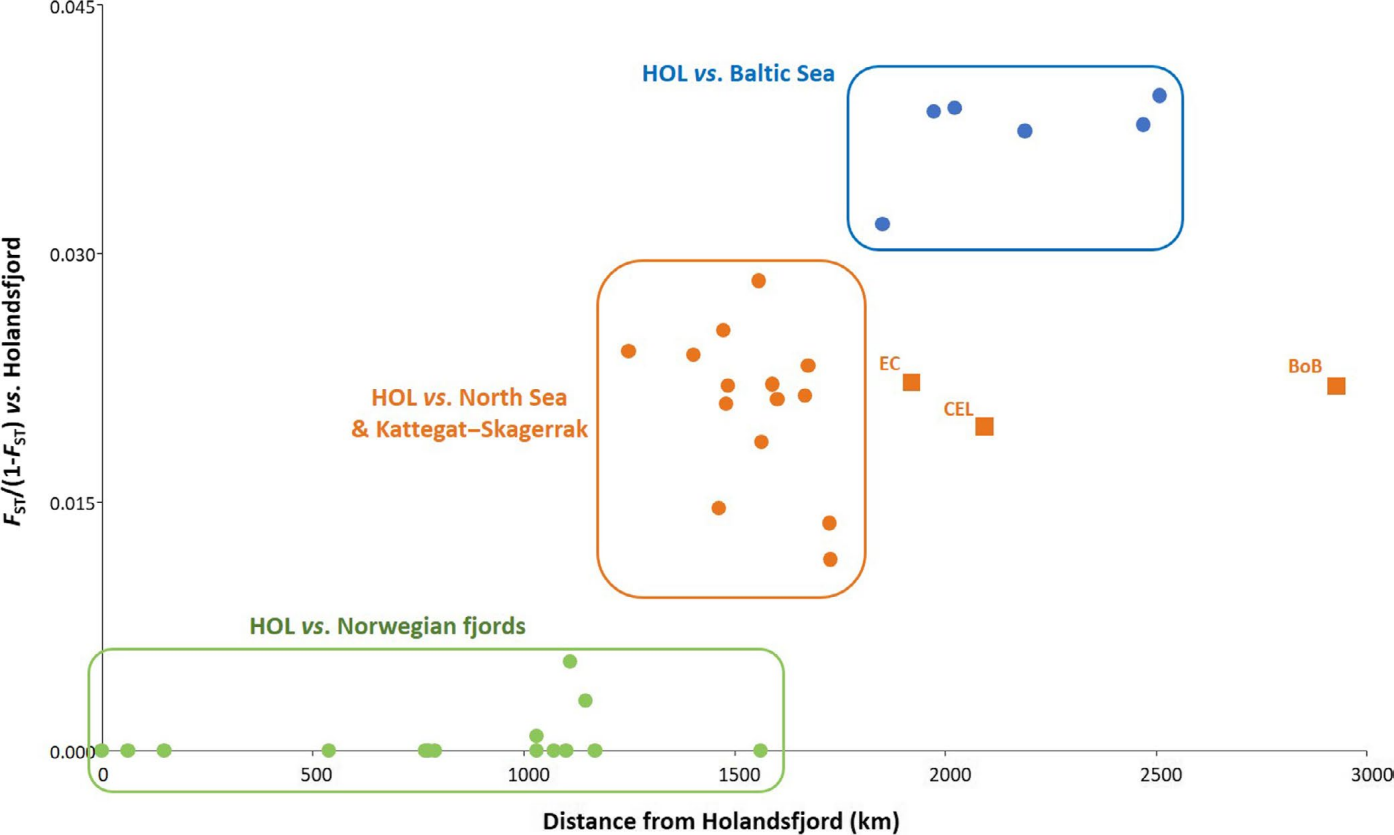
Plot of pairwise *F*_ST_/(1−*F*_ST_) between the northernmost location sampled in Norway (Holandsfjord—HOL) and each of the 37 remaining locations (excluding Adriatic Sea and Black Sea) versus the corresponding shortest water distance (in km). Plotted values fitted a regression with *R* ^2^ = .6145 and *p* < .0001. The colors correspond to the clusters in STRUCTURE, whereas the squares depict the relation between HOL and the sites in the English Channel (EC), Celtic Sea (CEL), and Bay of Biscay (BoB)

The shortest oceanic distance between the northernmost location sampled in Norway, Holandsfjord (HOL), and each of the 37 other locations (excluding the two out‐groups) significantly correlated with the corresponding genetic distance measured as *F*_ST_/(1−*F*_ST_): *R* ^2^ = .615, *p* < .0001; albeit, there was local variation over the studied geographic range (Figure 6). Hence, comparisons among the Norwegian fjord samples spanning a geographic stretch of some 1,500 km showed low *F*_ST_ across the board and no evidence of increasing genetic divergence with geographic distance. However, in a similar geographic span, when comparisons included Kattegat–Skagerrak and North Sea samples, an increase in genetic differentiation with distance from HOL was detected. Finally, the differentiation between HOL and the samples from the Baltic Sea plateaued around an average *F*_ST_ of 0.380.

**Figure 7:**
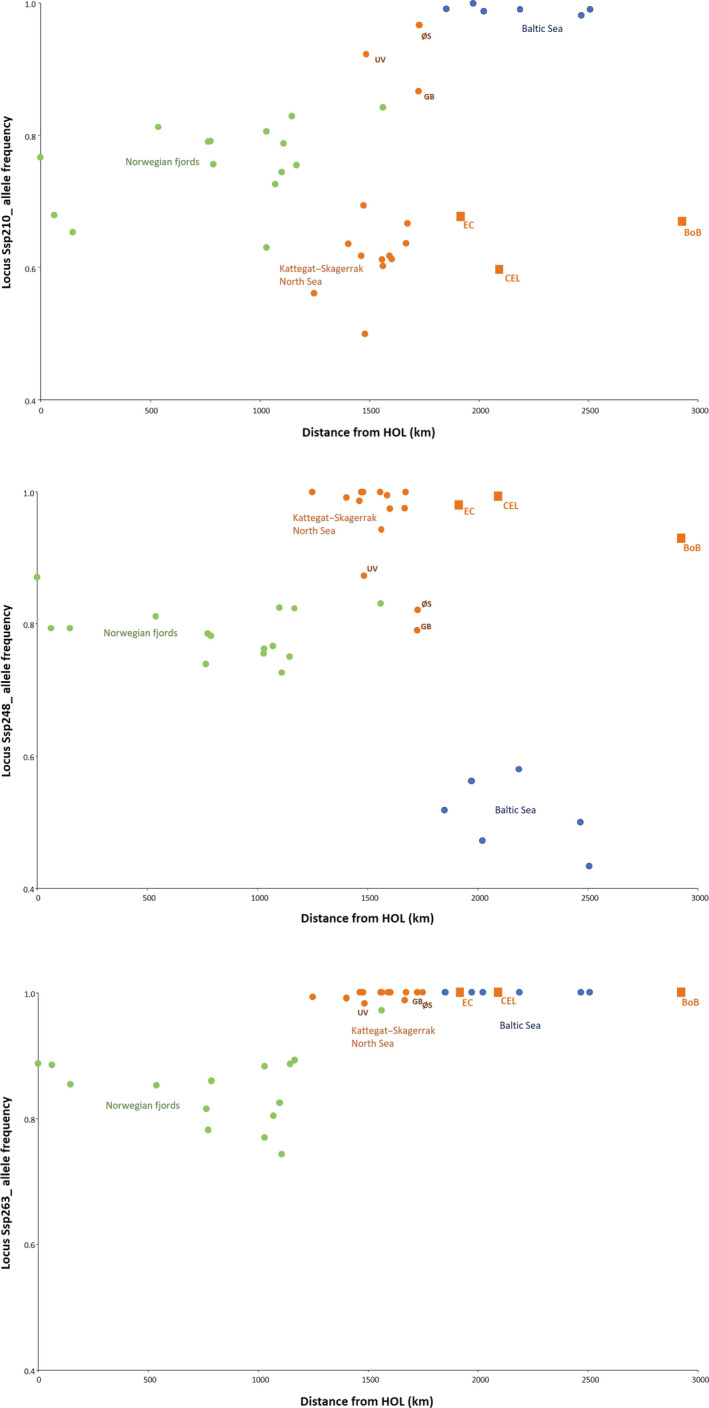
Allele frequency per sample as a function of the geographic distance to the starting point of the transect (HOL, in northernmost Norway) for the loci identified as undergoing divergent selection after consensus between LOSITAN and BayeScan. Sites are represented by dots colored corresponding to the patterns of the STRUCTURE barplots, whereas the squares depict the samples from the English Channel, Celtic Sea, and Bay of Biscay

Heat maps of the major allele frequency for neutral and outlier loci can be found in Tables S4 and S5, respectively, in Supplementary Information. For the three loci consistently flagged as outliers, pairwise allele frequency distances were also examined against the geographic distance. Locus Ssp210 (Figure 7a) and Ssp248 (Figure 7b) showed clear region‐specific differences among the three STRUCTURE groups. At locus Ssp210, Uddevalla, Great Belt, and Øresund occupied intermediate positions between the Norwegian and Baltic clusters and distant from the Kattegat–Skagerrak samples. However, at locus Ssp248, they clustered with the Norwegian samples. Conversely, allele frequencies at locus Ssp263 discriminated between the Norwegian populations and all the remaining ones (Figure 7c).

## 4 DISCUSSION

The primary goal of this study was to investigate population genetic structure of sprat in order to advise the ICES management plan for this species in the North Sea and its surrounding areas. To fulfill this aim, a suite of 91 SNP markers identified by ddRAD sequencing was genotyped in approximately 2,500 individuals collected from 40 locations. Three highly distinct genetic groups were identified, corresponding with the geographical regions: (a) Norwegian fjords; (b) the Northeast Atlantic region including the North Sea, Kattegat–Skagerrak, Celtic Sea, and Bay of Biscay; and (c) the Baltic Sea, including its transition zone with the North Sea, the latter exhibiting admixed genetic profiles. As the former ICES catch advice for sprat in Europe was given for stock units that partially mis‐align with the genetic data presented here, data from the present study have now been implemented by introducing a change in the stock units ICES uses for biological assessment of sprat (ICES, 2018c). Therefore, this study represents a case where novel genetic data on stock structure were directly used to inform relevant advisory bodies to align harvest regimes with biological units.

### 4.1 Patterns and underlying of population genetic differentiation

A handful of previous studies have contributed to the current understanding of sprat population genetic structure in spite of certain limitations regarding geographical scope, sampling coverage, and/or the resolution of the genetic tools implemented (Debes et al., 2008; Glover et al., 2011; Limborg, Hanel, et al., 2012; Limborg, Hanel et al., 2012). The results of the current study, which are based upon both a greater number of samples and genetic markers than all previous studies (91 genome‐wide SNPs in approximately 2,500 fish), largely aligned with former results but provide increased resolution. Primarily, the existence of three distinct geographically distinct genetic groups in the Northeast Atlantic was demonstrated, each of which showed pronounced lack of genetic differentiation within groups.

A striking result was the lack of genetic differences identified among Norwegian fjord samples spanning about 1,500 km coastline, suggesting extensive gene flow among fjords. Likewise, no statistically significant genetic differentiation was detected between samples from the Celtic Sea, the Bay of Biscay, and the North Sea, despite encompassing distances up to 2,000 km. Yet in contrast, several other comparisons indicated distinct genetic borders between areas separated by very short distances. For example, the sample from the Uddevalla fjord (UV, sample 16) showed clear divergence from the Kattegat samples collected offshore less than 40 km away. Likewise, the Norwegian west coast sample from Lysefjord (LYS, sample 14) showed clear divergence from samples from the North Sea, some of which were collected within distances of less than 250 km. This specific observation supports suggestions from an earlier study that there is little, if any, physical mixing and gene flow between sprat in Norwegian fjords and coastal areas, and sprat in the North Sea (Glover et al., 2011). Apart from Oslofjord, which showed low, albeit in some cases statistically significant genetic differentiation from fjords in western Norway, our sampling design did not include any of the several Norwegian Skagerrak fjord populations. We were therefore not able to determine whether population structure follows a gradient along the Norwegian Skagerrak coast. This needs to be investigated in future studies.

The lack of clear population genetic differentiation identified within each of the three main genetic groups, despite some samples within groups being separated by very large distances, coupled with the large genetic differences observed between groups, despite short distances between pairs of samples in some cases, begs the question: What are the mechanisms underpinning such distinct patterns in this small pelagic fish? In order to answer this question, different processes need to be considered. First, the relatively sharp genetic divergence observed from the Baltic Sea to the Kattegat and North Sea, coupled with the admixed genetic profiles of samples in the transition area, likely reflect a combination of demographic processes associated with Baltic Sea post‐glacial founder events, in addition to environmentally driven adaptations to Baltic Sea conditions (e.g., Momigliano et al., 2017). Strong genetic differences across the North Sea–Baltic Sea region, with admixed populations in the transition area, reflects the general pattern seen across a broad taxonomic range of species in this region (review in Johannesson & Andre, 2006). With increasing insights from genomic sequencing analyses, evidence is amassing for specific adaptations to brackish conditions (Berg et al., 2015; Lamichhaney et al., 2012; Limborg, Helyar, et al., 2012; Petereit et al., 2018; do Prado, 2018; Vilas, Bouza, Vera, Millan, & Martinez, 2010) and other environmental conditions specific to the Baltic Sea, such as light regime (Hill et al., 2019). It is possible that one or more of the SNPs identified as outliers in the present study may be located in genomic regions containing genes associated with adaptive processes to the environmental differences experienced in the gradient from the outer to inner Baltic. Candidate loci to divergent selection showed region‐specific allele frequency differences, as opposed to most of neutral loci (Tables S4 and S5), in agreement with the patterns found for the East Atlantic peacock wrasse (*Symphodus tinca*), endemic to the Mediterranean (Carreras et al., 2017). Significant allele frequency changes in genes that were differentially expressed after five generations of size‐selective harvesting have also been reported for zebrafish (*Danio rerio*) (Uusi‐Heikkilä, Sävilammi, Leder, Arlinghaus, & Primmer, 2017). However, disentangling demographic from adaptive effects on specific types of genetic variation typically requires genomic resources beyond those available in the present study.

The very low level of genetic differentiation observed among the samples collected from the Norwegian fjords, despite distances of up to ~1,500 km between them, may suggest that there is a high level of genetic and demographic connectivity among sprat in this region. Complex oceanic currents exist within and among Norwegian fjords, leading to retention in certain periods and flushing in others (Asplin et al., 2014; Asplin, Salvanes, & Kristoffersen, 1999; Johnsen, Fiksen, Sandvik, & Asplin, 2014). In turn, these complicated currents affect pelagic larval drift between fjords. However, knowledge of these currents does not provide us with data that would unequivocally enlighten our understanding of observed patterns in genetic connectivity across this region for this species. In addition, there is extensive evidence that sprat spawn in most, if not all, of the fjords along the Norwegian coastline (e.g., Bakken, 1973; Ellingsen, 1979; Torstensen, 1998). Furthermore, we cannot exclude the possibility that there is a low degree of genetic structure in this region eluding scrutiny with the set of markers used here. Effective population sizes of sprat are expected to be sufficiently large that genetic drift may be too low to render selectively neutral markers adequate for differentiating local demographic units (Gagnaire et al., 2015). Based on genome sequencing in another pelagic clupeid from the North Sea–Baltic Sea area, Atlantic herring (*Clupea harengus* L.), it was shown that the majority of the identified genomic variation exhibited no differentiation among populations that otherwise had strong genetic divergence for candidate genes inferred to be under local adaptation (Martínez Barrio et al., 2016). Studies like these emphasize that genetic marker‐based evidence for connectivity should be treated with some caution as future studies utilizing full‐genome tools may reveal currently unidentified divergence.

The observed lack of genetic structure of sprat along the Norwegian coastline contrasts with patterns in population genetic structure in other fishes in this region. For example, demersal Atlantic cod (*Gadus morhua* L.) display a north–south genetic gradient (Dahle, Quintela, et al., 2018), while rocky shore wrasse species such as corkwing (*Symphodus melops* L.) and ballan wrasse (*Labrus bergylta* A.) show clear differentiation across a sandy stretch of habitat discontinuity (Blanco González, Knutsen, & Jorde, 2016). Clearly, species with different environmental requirements, dispersal mechanisms, and life‐history strategies display very different patterns of genetic structure in this region (e.g., André et al., 2016; Florin & Höglund, 2008; Knutsen et al., 2018). In a broader context, pelagic species such as the European sardine, *Sardina pilchardus*, Walbaum 1792 sampled from NE Spain to south of Morocco reflected a single evolutionary unit with mtDNA yet showed weak but significant genetic differentiation depicting an IBD pattern when using microsatellites (González & Zardoya, 2007). In contrast to sardine and in line with our results, the sprat congener *Sprattus fuegensis* (Jenyns 1842) in Patagonian Chile display two highly differentiated genetic clusters, potentially the result of larval retention via combination of oceanographic mesoscale processes combined with local geographical configuration (i.e., embayment areas, islands, archipelagos) (Canales‐Aguirre, Ferrada‐Fuentes, Galleguillos, & Hernández, 2016).

Loci under divergent selection can be applied as an efficient tool to detect population structure in marine species showing high dispersal and gene flow coupled with low genetic drift (e.g., Nielsen et al., 2012). In the present study, some 10% of the analyzed loci were candidates for divergent selection although genome‐wide markers combined with phenotypic or environmental variation would be required to identify the underlying causative forces. For instance, observations from other highly mobile marine organisms coupling outlier loci with adaptive variation showed many genomic regions displaying elevated divergence, apparently as a response to temperature‐ and salinity‐related natural selection in Baltic Sea herring (Guo, Li, & Merilä, 2016; Limborg, Helyar, et al., 2012). Similarly, environmental conditions are suggestive of driving adaptive selection in other clupeids such as the European anchovy, *Engraulis encrasicolus* L. Hence, geographic gradients in sea temperature, salinity, and dissolved oxygen in the Adriatic Sea appear to promote adaptive differences in spawning time and early larval development among populations (Ruggeri et al., 2016). Furthermore, Catanese et al. (2017) showed, using 96 SNPs derived from genomic and transcriptomic data, that the selective pressure related to river mouths apparently acts on the same genes in the Atlantic Ocean as well as in the Tyrrhenian Sea and North Adriatic Sea. These SNP outliers were also associated with salinity variability or involved in a critical stage of fertilization process.

### 4.2 Potential mixing in Kattegat–Skagerrak and the western Baltic Sea

There was no sign that samples collected in offshore Skagerrak–Kattegat areas at any time of the year contained more than a single genetic group, and there was hence no evidence of more than one stock (mixed stocks), as is the case for another clupeid feeding in the same area, Atlantic herring (Bekkevold et al., 2015a). Although distinct genetic differentiation was observed between samples from the edges of each of the main genetic clusters, evidence of physical mixing and genetic admixture was observed in Swedish Skagerrak fjords (typified by Uddevalla) and the Belt Sea (Great Belt and Øresund) located at the southern border of the Kattegat (Figures 3b and 4). Although the combination of observed genotypes and North Sea x Baltic Sea hybrids created in silico suggests admixture (i.e., gene flow) in this region as reflected in Figure 5 and Figure S2, we cannot exclude the possibility that this also reflects physical mixing of fish from the main genetic groups. The latter is explained due to the approximately 20% of the individuals displayed non‐admixed patterns. Furthermore, physical mixing between the main genetic groups is likely to show spatiotemporal variation in regions such as Skagerrak (Weist et al., 2019) and the western Baltic Sea (ICES, 2018a, 2018b, 2018c, 2018d, 2018e, 2018f), as demonstrated in other marine fishes (e.g., Bekkevold et al., 2015b; Hemmer‐Hansen et al., 2019; Knutsen et al., 2018). Detailed temporal sampling in these areas, ideally combined with biometric and/or life‐history measurements (Moore et al., 2019), is recommended to further elucidate the physical movement or genetic admixture patterns in both Kattegat and Skagerrak where all three genetic groups may converge. Certainly, in order to advise fishery efforts in this region, such analyses should be a priority.

### 4.3 Management implications

While questions remain, regarding the extent of genetic admixture and physical mixing among the three major genetic groups in time and space, especially in the coastal Kattegat–Skagerrak areas, our data provide a clear overall picture of population genetic structure for sprat. As a direct consequence of our genetic analyses, together with other biological evidence (ICES, 2018b), the stock definitions currently in place when assessing spawning stock biomass have now been modified to consider sprat in the North Sea and Kattegat–Skagerrak area as a single management unit (ICES, 2019). Thus, our study contributes to an increasing list of successful implementations of fisheries genetics in assessment and management (see Dahle, Johansen, et al., 2018).

### 4.4 Future perspectives

The SNPs used in the current study were developed upon a sample of eight individuals from one of the Norwegian sites. These low sampling numbers allowed to confirm that alleles were robustly analyzable although hampered any reliable estimate of allele frequencies. However, the primary objective was to identify a “random” panel of markers for high‐throughput genotyping to investigate the population structure of this species, without aiming for diagnostic or geographically informative SNPs. The suite of 91 SNPs genotyped on approximately 2,500 individuals allowed to successfully identify three highly distinct genetic groups, corresponding with the following geographical regions: (a) Norwegian fjords; (b) the Northeast Atlantic region including the North Sea, Kattegat–Skagerrak, Celtic Sea, and Bay of Biscay; and (c) the Baltic Sea. Therefore, considering the wide‐ranging MAFs between geographic groups that loci displayed, and that population genetic structure showed plausible geographic and biologic resolution, ascertainment bias seemed not to be of major concern. However, although this simple marker‐identification procedure suitably matched our objectives, whole genome‐based approaches specifically looking for outlier loci and signs of adaptation may lift knowledge further in the future by capturing variation that might have eluded our scrutiny so far.

A geographically broad and dense sampling design is beneficial for any population genetic study. Here, a denser net of samples in the southernmost part of Norway as well as in the North Sea and Kattegat–Skagerrak areas might help outlining the transition zone with higher precision. Furthermore, including samples from the meridional range of the distribution and increasing the density in areas such as British Isles, western French coast, Mediterranean, and Black Sea would provide a comprehensive picture of the number of populations and level of connectivity between them in this species.

## CONFLICT OF INTEREST

None declared

## Supporting information

Fig S1‐S4

Table S1‐S2

Table S3

Table S4‐5

## Data Availability

The suite of SNP loci and with their corresponding flanking regions, together with the raw data, is available in Supplementary Information_Raw data, Tables S1 and S2, respectively.
