# Supplementary material for: Genetic analysis redraws the management boundaries for the European sprat
Source: Evol Appl. 2020 Mar 17;13(8):1906–22. doi: 10.1111/eva.12942 (PMC7463317; doi:10.1111/eva.12942)
Supplement: Supplementary file 1 — Fig S1‐S4 [file EVA-13-1906-s001.docx]

**SUPPLEMENTARY INFORMATION: FIGURES**

|  |
| --- |

**Fig. S1.-** Discriminant analysis of principal components (DAPC) for the forty total sprat sampling sites: representation of axis 2 and 3.

| a)   |
| --- |

| b)   |
| --- |

| c)   |
| --- |

**Fig. S2.-** Loading plots of loci on axis 1 (a), 2 (b) and 3 (c) of the DAPC for the 40 sampling sites genotyped at 91 SNPs.

| a)  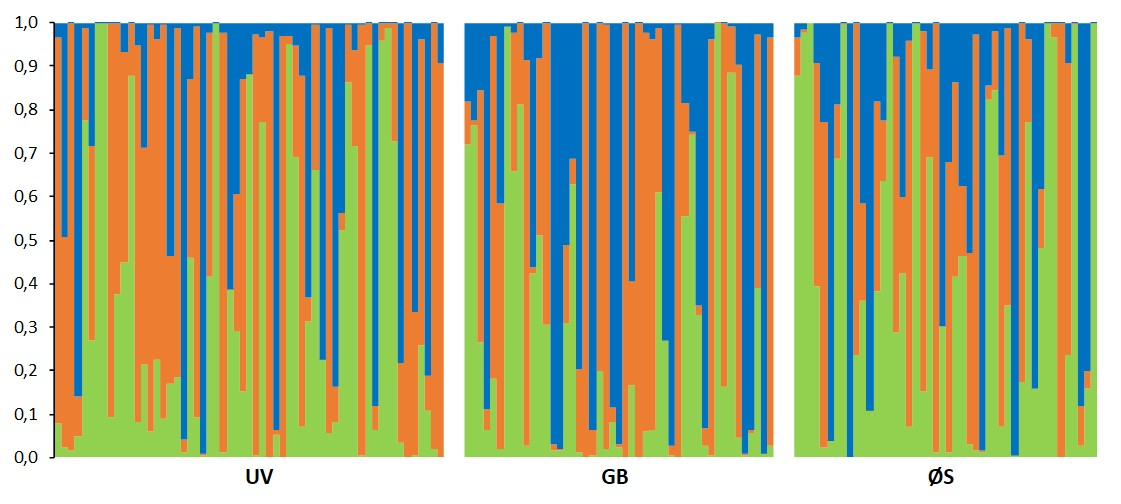 |
| --- |
| b)  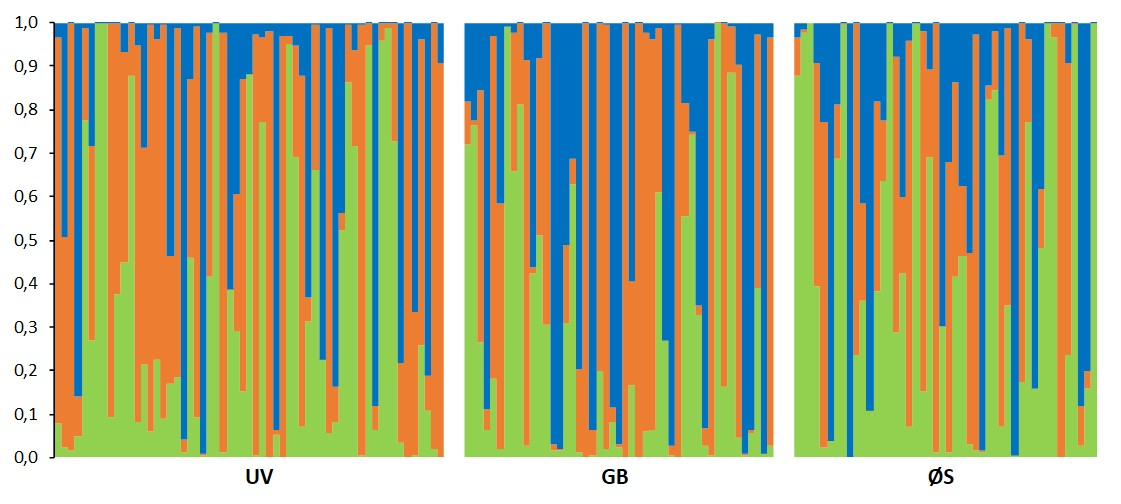 |

**Fig. S3.-** Comparison between the STRUCTURE barplot (a) corresponding to the contact zone (UV, GB, ØS) and the assignment probabilities to the three main STRUCTURE genetic clusters obtained through ONCOR (b) for the same individuals sorted in identical order.

**Fig. S4.-** Discriminant analysis of principal components (DAPC) for the sprat sampling sites without outgroups assessed by 81 neutral markers.
